# A systematic review of smoker and non-smoker perceptions of visually unappealing cigarette sticks

**DOI:** 10.18332/tid/82191

**Published:** 2018-01-31

**Authors:** Aaron Drovandi, Peta-Ann Teague, Beverley Glass, Bunmi Malau-Aduli

**Affiliations:** 1 College of Medicine and Dentistry, James Cook University, Townsville, Australia

**Keywords:** public health, social medicine, smoking appeal, cigarette attributes

## Abstract

**INTRODUCTION:**

Cigarette stick appearance can significantly contribute to perceptions of cigarette taste, harm, and appeal, and may be modified to reduce positive perceptions of cigarettes and other tobacco products. A systematic review was conducted to investigate how smokers and non-smokers identify cigarettes as being attractive or unattractive, and the resulting perceptions of cigarette appeal, perceived harm, and impact on quit intentions.

**METHODS:**

Eligible articles were identified using database searches conducted with a date range of January 1990 to May 2017 in PubMed, CINAHL, PsycINFO, Google Scholar and Web of Science. Articles were included if they evaluated participant (any smoking status) perceptions of visual cigarette stick attributes. We identified studies describing visual attributes of cigarette sticks and the resulting perceptions of participants. Changes or differences in quitting intentions, cigarette appeal, perceptions of taste, and cigarette harm, and the likelihood of smoking uptake were recorded. Data were grouped into two main categories: those of physical cigarette design, and those including health messages on cigarette sticks.

**RESULTS:**

Of the 950 identified non-duplicated records, 9 matched the eligibility criteria. These studies were all conducted in developed countries, and largely enrolled adolescent and young adult smokers and non-smokers. Slim, lighter coloured and branded cigarettes were favoured over longer, broader, or darker coloured cigarettes, and those without any branding or embellishments. Health warnings including ‘Minutes of life lost’, ‘Smoking kills’, and the names of carcinogenic constituents in cigarettes, reduced cigarette attractiveness and increased participant quit intentions.

**CONCLUSIONS:**

Cigarette appeal and resulting smoking behaviours can be influenced by several visual attributes of individual cigarettes. Unappealing visual attributes of cigarette sticks, including modifications to the size and colour of cigarettes, and the inclusion of health warnings on cigarette sticks may serve as an effective tobacco control method, potentially leading to a reduction in tobacco use.

## INTRODUCTION

Public education on the dangers of tobacco use is an integral component of the World Health Organisation’s (WHO) Framework Convention on Tobacco Control (FCTC) guidelines^1^, with over 100 countries having implemented the mandatory inclusion of written health warnings and graphic images on the packaging of all tobacco products^2^. These interventions have affected perceptions of the harm caused by tobacco and increased quit attempts by smokers, leading to health benefits for the smoker and their community^3-5^.

The more recent implementation of plain/standardised packaging of tobacco products occurred in late 2012 in Australia, in 2016 in France and the UK, and in early-mid 2017 in Hungary and Norway, with New Zealand and Ireland also planning to implement these change in 2018. The removal of branding colours and imagery increases the prominence of written and illustrative health warnings; this has led to a reduction in the prevalence of smoking amongst Australians, and is hoped that it will achieve the same result in the countries that adopt plain packaging^6^. Plain packaging is also expected to reduce false perceptions of cigarette harm and minimise the effects of brand appeal, which is particularly important to protect youth and young adults^6-11^. These changes also improve smokers’ awareness of the harms of smoking, which can be negated by the presence of appealing colours and other persuasive aspects of tobacco packaging and branding^12-13^. Plain packaging legislation has also affected individual cigarette appearance, which is to be either all-white or white with a cork tip^14-16^.

Tobacco manufacturers expend significant resources into identifying the most appealing combination of cigarette stick and packaging features to distinguish their products from competitors and ensuring brand loyalty, which is often attained early during the life of a smoker^17-19^. Notable physical aspects of cigarettes include length, diameter, filter, colouration, patterns, and textual messages. Modifying these attributes may negate the persuasive methods employed by tobacco manufacturers who have designed cigarettes to appeal to segmented populations according to their psychological and psychosocial needs, such as young women who prefer slim designs and white colouration^17-19^. Conversely, invoking negative perceptions towards tobacco products, through the use of dissuasive methods and making it harder for smokers and non-smokers to avoid or ignore the intended health messages, may therefore encourage quit attempts amongst smokers and help non-smokers to refrain from initiating smoking.

The objective of this systematic review is to consolidate current research evaluating smoker, non-smoker, and ex-smoker perceptions of various visual cigarette stick attributes. The findings of this review may direct further research into devising methods to deter smokers and non-smokers from using tobacco products.

## METHODS

The Preferred Reporting Items for Systematic Reviews and Meta-Analyses (PRISMA) statement was used as a reporting guide for this systematic review^20^.

### Study selection

Articles were eligible for inclusion if they were in English, original-research papers, and gathered participant (any smoking status) perceptions of visual cigarette stick attributes. Articles that reported modifications to cigarette packaging alone or perceptions of non-visual cigarette attributes were excluded from this review.

### Data sources

Published articles were identified through electronic searches from January 1990 to May 2017 in PubMed, CINAHL, PsychINFO, Google Scholar, and Web of Science. Search terms included the following combinations: ‘cigarette stick warning’, ‘novel cigarette warning’, ‘tobacco health warning’, ‘cigarette stick perceptions’, ‘cigarette label warning’, ‘cigarette novel packaging’, ‘dissuasive cigarette’, ‘cigarette health labelling/labeling’, and ‘tobacco warning labelling/labeling’. Titles were read to identify potentially relevant articles, and we initially included any article that appeared to contain modifications to either tobacco packaging or cigarette sticks. Abstracts were reviewed, and articles discussing only modifications to tobacco packaging were subsequently excluded, while articles discussing changes to cigarette sticks were retained for full article review. Eligible articles had their reference lists searched to identify additional articles for inclusion.

### Synthesis of results

Data extracted included: basic study characteristics (sample sizes, gender and age distribution, participant smoking status, and location of participants), types of cigarette stick presented and their relevant visual attributes, and the resulting perceptions of participants. The primary outcomes for this review are: the effect of visual cigarette stick attributes on cigarette appeal and expected strength of taste, the resulting perceptions of cigarette harm, changes in quit intentions, and the likelihood of smoking initiation. Data were grouped into categories of cigarette attribute: those involving physical design changes (including changes in length, colouration, diameter, and embellishments), and those using written or illustrative health messages.

### Quality appraisal

Eligible studies were assessed for quality, using the Critical Appraisal Skills Programme (CASP) Qualitative Research Checklist and the Joanna Briggs Institute Critical Appraisal Checklist for Qualitative Research^21-22^. These two checklists assess study clarity and appropriateness relative to the aims and objectives listed, the methodological processes used, the appropriateness of collection and representation of data, and the clarity of representation of findings and conclusions. Two of the authors (AD and BM-A) independently assessed all eligible studies for quality. A score of at least 8 out of 10 in both checklists resulted in articles being considered as high quality, at least 6 out of 10 as medium quality, and 5 or less as low quality.

## RESULTS

### Study characteristics

**Figure 1:**
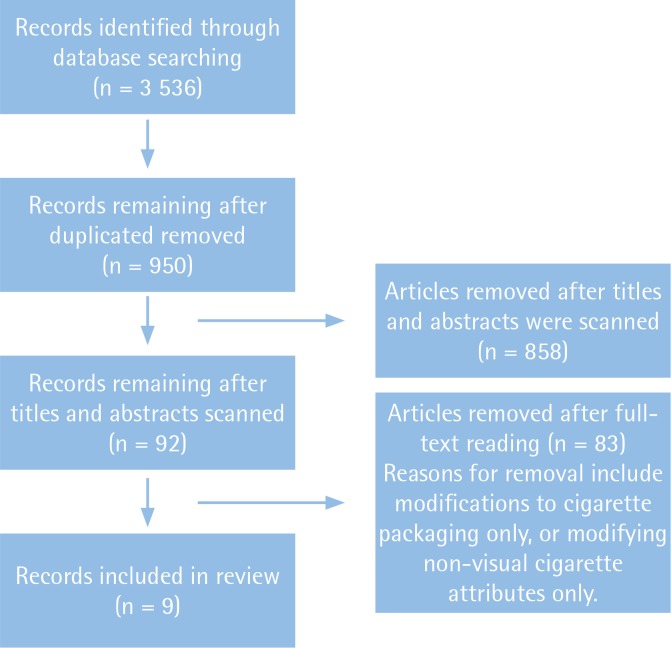
Flow chart of systematic literature search

Figure 1 illustrates the article selection process. The search strategy initially identified 3 536 articles, which were reduced to 950 after duplicates were removed. After scanning titles and abstracts, a further 858 articles were removed, leaving 92 articles that appeared to discuss either cigarette packaging or cigarette stick modifications. Having completed a full article review, a further 83 articles were removed, the most common reason being that these articles only evaluated the effects of cigarette packaging and did not consider cigarette sticks. This resulted in nine studies that met the inclusion criteria for this review. Table 1 details study and participant characteristics, and quality scores. A secondary search was conducted, owing to the low number of eligible articles, employing Boolean operators on words such as cigarette, tobacco, smoking, stick, label, labelling, and warning. No additional eligible articles were found.

**Table 1:** Study characteristics and participant demographics for eligible articles

*Author and Study Year*	*Year conducted*	*Participant numbers*	*Study country*	*Study design*	*Gender % (m : f)*	*Age range (years)*	*Smoking status of participants*	*Quality score#*
**Borland and Savvas (2013)****^23^**	2011	160	Australia	Online survey	50 : 50	18 - 29	80.6% active smokers^&^ 19.4% ex-smokers	JBI 8 CASP 10
**Ford et al. (2014)****^24^**	2011	48	Scotland	Focus groups	50 : 50	15	19% active smokers* 10% ex-smokers 71% never smoker	JBI 9 CASP 8
**Hoek and Robertson (2015)****^25^**	2011	9 13	New Zealand	Focus groups interviews	0 : 100	18 - 25	100% active smokers (45% daily smokers)	JBI 9 CASP 8
**Moodie et al. (2015a)****^26^**	2013	75	Scotland	Focus groups	0 : 100	12 - 24	32% occasional smokers 68% non-smokers	JBI 7 CASP 7
**O’Connor et al. (2015)****^27^**	2011	1 220	USA	Interviews	55 : 45	18 - 35	48.3% active smokersα 28.9% ex-smokers 22.8% never smoked	JBI 7 CASP 7
**Hoek et al. (2015)****^28^**	2014	313	New Zealand	Online survey	49.5 : 50.5	18+	79.5% daily smokers 20.5% social smokers	JBI 8 CASP 8
**Hassan and Shiu (2015)****^29^**	2012	88 120	Scotland Greece	Interviews	39 : 61 60 : 40	86% <30 80% <30	100% smokers 100% smokers	JBI 4 CASP 5
**Moodie et al. (2015b)****^30^**	2012	49	Scotland	Focus groups	0 : 100	16 - 24	100% smokers*	JBI 7 CASP 7
**Moodie et al. (2016)****^31^**	2014	1 205	UK	Interviews	50 : 50^	11 - 16	~21% smoker^ ~79% never smoker^	JBI 7 CASP 7

# Both the JBI and CASP quality appraisal checklists have maximum scores of 10

& Of the active smokers, 10.1% stated that they smoked less than daily

* Definition of smoking in these studies was smoking at least once per week

α Definition of a smoker in this study was smoking within the past 30 days

^ Gender distribution and smoking status were not specifically stated, though data described allowed an estimation

Of the 9 studies included, 4 were identified as having high quality, 4 as moderate quality, and 1 as low quality. Checklist items commonly not addressed were the cultural or theoretical background of the researchers, and the potential influences of the researchers on the participants and vice versa. There were no disagreements between the two reviewers on the quality scores for any study. Studies investigating only participant perceptions of capsule cigarettes were excluded from this review. The authors believe that the unique packaging and flavouring aspects of these products would have confounded the assessment of participant perceptions of visual-only attributes.

Table 2 contains a summary of the cigarette attributes evaluated and the analytical methods used within each eligible study. Five studies evaluated perceptions of the physical design aspects of cigarette sticks, including variations in cigarette length, diameter, colouration, and branding. Three studies evaluated perceptions of health messages on cigarette sticks, and one study evaluated both physical design attributes and health warnings on cigarette sticks. Four studies used computer-generated images or photographs of cigarettes to gather quantitative data through online surveys or interviews, identifying how differences in cigarette appearance affected rankings of appeal, quit intentions, and intent to purchase. Three studies used locally available or modified cigarettes, and one study used photographs of cigarettes to invoke open discussions with participants on their perceptions, and one study employed a mixed-methods approach using modified cigarettes in one-on-one interviews, to gather participant perceptions of cigarette taste, harm, and appeal.

**Table 2:** Summary of interventions, participant tasks, and analytical methods used within each included study

*Study*	*Cigarettes presented*	*Cigarette modification*	*Control cigarettes*	*Modified cigarettes displayed to participants*	*Participant task(s) and discussion(s)*	*Analytical methods*
**Borland and Savvas (2013)****^23^**	Computer generated	Physical design	White with cork filter	Fourteen cigarettes, including: slim, short, extra-long, embellished, and branded	Ranking of attractiveness, quality, and taste	ANOVA (SPSS)
**Ford et al. (2014)****^24^**	Locally available	Physical design	None stated	Eight cigarettes with combinations of varied length, diameter, brands, and colour	Open discussions, followed by ranking of attractiveness, strength and perceived harm	Thematic analysis (NVivo)
**Hoek and Robertson (2015)****^25^**	Photographs	Physical Design	None stated	Twenty cigarettes with white, tan, bright, or dark colours on the stick and filter	Discussions on cigarette attractiveness and health risks of cigarette use	Thematic analysis (NVivo)
**Moodie et al. (2015a)****^26^**	Locally available	Physical design	White with cork filter	Eleven cigarettes of varied diameter, length, colour, branding, and embellishments	Ranking of cigarette appeal, taste, and harm	None
**O’Connor et al. (2015)****^27^**	Modified cigarettes	Physical design	None stated	Three cigarettes: one shorter without a filter, and two filtered king size, one with a white filter and one with a cork filter	Perceptions of cigarette appeal, taste, and harm	χ^2^, logistic regression (SPSS)
**Hoek et al. (2015)****^28^**	Computer generated	Physical design and health messages	White with cork filter	Five cigarettes: one clean white, one with ‘Smoking Kills’, one with ‘Minutes of life lost’, and two with dissuasive colours	Intent to purchase, and cigarette appeal	ANOVA, t-tests (SPSS)
**Hassan and Shiu (2015)****^29^**	Photographs (Scotland) Modified (Greece)	Health messages	White with cork filter (Scotland only)	One cigarette listing five toxic cigarette constituents (Scotland only) and one with ‘Minutes of life lost’ (Scotland and Greece)	Rating of cigarette attractiveness (Scotland) Quit intentions (Scotland and Greece)	χ^2^, ANOVA, t-tests (SPSS)
**Moodie et al. (2015b)****^30^** **[30]**	Modified cigarettes	Health message	None stated	Four cigarettes with ‘Smoking Kills’ in a variety of positions	Open discussions on perceptions of cigarettes	Thematic analysis
**Moodie et al. (2016)****^31^**	Photograph	Health message	White with cork filter	One cigarette with ‘Smoking Kills’ printed in red on its shaft	Perceived efficacy of warning and perceptions of warnings on cigarettes	χ^2^, logistic regression (SPSS)

### Study findings

The findings of the nine eligible studies have been grouped under two main headings. The first section of the results discusses participant perceptions of physical cigarette design, and details responses to modifications in cigarette length, diameter, and colour. This includes the presence of coloured bands, logos and embellishments. The second section discusses perceptions of health warnings on cigarettes, and details the responses given by participants towards the inclusion of textual messages either on the filter or shaft of cigarettes. Three health messages were evaluated: ‘Smoking kills’, ‘Toxic constituents’, and ‘Minutes of life lost’. Differences in participant perceptions related to smoking status or demographic identities have been clarified throughout the results, if included in each eligible article.

### Perceptions of physical cigarette design

Borland and Savvas^23^ found that physical appearance, embellishments, and the branding of cigarettes significantly affected their attractiveness, perceived quality, and perceived strength of taste. The standard dimension cigarette received the best attractiveness, quality, and choice preference scores amongst smokers. Gold banded and branded cigarettes were also found to be more favourable to smokers, compared to white or blue tipping, or unbranded cigarettes. Men viewed the slim cigarettes less favourably than women, with women also more strongly associating cigarette attractiveness with quality^23^.

Ford et al.24 found that any cigarette not aligned with the participants’ opinion of ‘standard’ received significant attention. Unlike the responses reported by Borland and Savvas23, these younger participants felt that the slim and super-slim cigarettes were more ‘cool’ and ‘fancy’, less harmful than the larger cigarettes, and scored the highest ratings for attractiveness. The cigarettes considered as ‘standard’ were seen to be the most ‘plain’ and ‘boring’, and more closely linked with the stigma associated with smoking. Decorative branding and brighter colours also received more positive ratings amongst participants, whereas the longer length brown cigarettes were seen as ‘boring’ or ‘cheap’ and more unpleasant than the lighter coloured cigarettes. Lastly, there were mixed reactions to the king-size white-tipped cigarettes, as the larger size was associated with stronger taste and being more harmful, though the white colouration had the opposite effect^24^.

The focus groups in Hoek and Robertson25 indicated that white cigarettes were more strongly associated with freedom of choice, financial superiority, and a higher social status. Tan coloured cigarettes were instead associated with a lack of discretion and with ‘stereotypical’ addicted smokers who experience more social discrimination. Cigarettes that were more brightly coloured (silver, bright red, and lilac) were found to be attractive and possibly assisted in avoiding social stigma, and supported differentiation from ‘stereotypical’ smokers who are normally seen as being distasteful or unhealthy^25^.

The in-depth interviews conducted in this study found that although some smokers stated that they would smoke cigarettes irrespective of the colour; though the darker colours were generally associated with poor health, sickness and more likely to motivate cessation attempts. The participants indicated that these dark colourations opposed their desire to appear ‘innocent’, ‘clean’, and ‘sophisticated’ whilst smoking, attributes more strongly aligned with the white cigarettes^25^.

Similar results were found by Moodie et al.^26^, with the pink-coloured cigarette, in particular, receiving a largely positive response from both non-smokers and occasional smokers, by being regarded as ‘young’, ‘fun’, ‘pretty’, and encouraged an interest in smoking amongst participants. This effect was strengthened by the perception that the pink cigarette would have a more pleasant taste, and would cause less harm compared to the other cigarettes presented. Similar responses were also given towards the black aromatised cigarette (which included a gold band), with its unusual colour piquing interest and appeal, and giving it a sense of ‘class’, though occasional smokers had mixed reactions to the black cigarette^26^. Unlike the pink cigarette however, the black was perceived to imply a stronger taste and greater level of harm. The aroma of the black cigarette also strengthened its appeal, with participants likening it to ‘liquorice’ or a ‘candle’. Non-smokers generally found cigarettes to be less appealing than occasional smokers. Responses towards the slimmer cigarettes in this study agreed with those reported in the study by Ford et al.^24^, where their appeal was generally rated high in comparison to ‘standard’ cigarette appearance. This was largely due to perceived discretion, and a sense of reduced strength, better taste/flavour, and reduced harm. Some participants mentioned that these cigarettes would likely contain less harmful ingredients, and may be a suitable option for those who want to quit, smoke casually, or are just initiating smoking. Decorative designs and logos on these slim cigarettes also enhanced their appeal, making them appear ‘cute’ or ‘cool’^26^.

The largest study included in this review by O’Connor et al.^27^ found that the two cigarettes with filters were generally received more positively than the cigarettes without a filter, despite being shorter, which led to higher appeal ratings in the earlier research by Ford et al.^24^. The cork-tipped cigarette was considered the most attractive, and perceived to have the best taste, and was the most favourable to try, despite that nearly half of the participants expected the white tipped cigarette to be the least dangerous of the presented cigarettes. Compared to never smokers, current smokers were more likely to choose the cork and white-tipped cigarettes, whilst men and ex-smokers were most likely to choose the cork-tipped cigarette. The cigarette without a filter was considered to be the most dangerous, and received the lowest rating for willingness to try, with most smokers perceiving a decrease in potential harm from the included filters^27^.

Hoek et al.^28^ used a ‘Best-Worst Choice’ model, where participants indicated which cigarettes they would most or least likely to choose, based on the images presented. The cigarettes intentionally designed to be unappealing with dissuasive colours were less likely to be selected by respondents than the standard (brown tip) or feminine (white tip) cigarettes, and were significantly less appealing compared to the standard cigarette^28^.

### Perceptions of health messages on cigarettes

Hoek et al.^28^ also evaluated participants’ perceptions of health warnings on cigarettes. The ‘Minutes of life lost’ cigarette was the least selected, and had the lowest appeal rating amongst participants, due to its blunt and morbid message. The responses were assessed by type of smoker (daily or intermittent), gender, ethnicity, and age. Intermittent smokers had lower ratings of cigarettes with warnings on them, whereas daily smokers were more affected by cigarette colour. Women had lower ratings for all dissuasive cigarettes, whilst men had a lower average rating for the standard and the feminine cigarettes. Maori/Pacific participants reported lower ratings for most dissuasive and feminine cigarettes. Age appeared to increase negative ratings for all dissuasive cigarettes, with highest negative ratings for the older than 55 year olds, followed by the 35-54 year olds, in contrast to the 18-34 year olds^28^.

The Scottish participants in the study by Hassan and Shiu^29^ showed significant differences in cigarette attractiveness between the intervention and control groups, though there was no significant difference in attractiveness when comparing the two intervention cigarettes. Post-exposure quit intentions were also significantly different, including those between the two intervention cigarettes, with the ‘Minutes of life lost’ cigarette eliciting a greater increase in quit intentions compared to the ‘Toxic constituents’ cigarette. The Greek participants in this study corroborated these results, reporting significant increases in post-exposure quit intentions, after being given the ‘Minutes of life lost’ cigarette to hold^29^.

Moodie et al.^31^ found that some participants viewed the ‘Smoking kills’ messages as being ineffective, due to the warning already being present on cigarette packaging. However, many participants indicated that the constant display of the health message whilst smoking served as a persistent reminder of the harms of smoking, as well as creating a perceived reduction in social standing. The location of the health warning also influenced participant responses, with some participants stating that they could easily obscure the warning if it was placed only on the filter. However, others thought placement on the filter would result in a prolonged duration of exposure, being visible in ashtrays (or elsewhere) after the cigarette has been discarded, serving as a constant reminder for other viewers. The optimal position identified for placement was down the length of the cigarette paper, which was considered in the subsequent study by Moodie et al.^30,31^.

Most participants in Moodie et al.^31^ (especially never smokers) viewed health warnings on cigarettes as being effective in preventing people from initiating smoking, and they thought that such warnings would prompt smokers to quit smoking. Most participants, including half of current smokers, also supported the inclusion of warnings on all manufactured cigarettes^31^.

## DISCUSSION

Altering the appearance of tobacco packaging, through the inclusion of health warnings, graphic images, and plain packaging, may have contributed in reducing the health and financial impacts of tobacco use^2,4,5,32^. However, some researchers argue that as the cigarette stick is the item that is actually consumed when smoking, this form of public health intervention would be of greater or additional benefit^28,31,33^. This review demonstrates the influence of cigarette stick appearance on cigarette appeal, perceived strength of taste and harm, quit intentions, and likelihood of initiating smoking.

Identified responses to cigarette appearance include: feelings of social standing, sophistication, perceived quality, pleasurable effects, and level of harm associated with smoking. Modifications to cigarette appearance that trigger a reduction in persuasive and an increase in dissuasive visual attributes can potentially reduce the attractiveness of cigarettes, leading to an increased likelihood of cessation attempts^16,18,25,29,31,32^.

Dissuasive cigarette sticks are thought to disrupt the intended persona of smokers, weaken the distinctive attributes that smokers seek, and lessen the appeal of smoking to non-smokers^28^. Distinctive attributes, such as high social standing, are achieved through long-term loyalty to a brand considered to be of high quality^28^. Tobacco research and marketing into persuasive attributes of cigarettes and their packaging, as evidenced by internal tobacco manufacturer research, has led to significant cultural acceptance and admiration towards smoking, even having a residual influence within countries with strict regulations on tobacco advertising^6,17-19,34-37^. While these perceptions of smoking have diminished over time, there are still inaccurate perceptions of cigarette appearance, such as the perception of slim, white-tipped, and embellished cigarettes as being of increased quality and reduced harm^24,26,28^. Several low and middle income countries have fewer restrictions on tobacco product marketing and advertising, as shown by Smith et al.^38^, with over 3200 (99.75% of sampled) cigarette sticks from 14 countries sporting decorative colours and designs, with these attributes thought to convey luxury, femininity, and reduced cigarette harm^38^.

Two types of cigarette attributes were investigated in this review; those involving differences in cigarette dimension or colouration, and those involving the addition of health warnings on cigarettes. Although the studies demonstrated the potential public health benefits of implementing visually unappealing cigarettes, it must be noted that global generalizability of the results could be affected by the limited number of nationalities included in this review^16,25,28-30^. The countries represented have different levels of tobacco control policies, likely affecting the general perceptions of their respective populations towards tobacco products^39^.

Familiarity was a strong factor for cigarette attraction, with the modified appearance of cigarettes thought to disrupt cue consistency and expectations, invoking dissonance amongst smokers, particularly established smokers^16,25,28^. Responses from young, female smokers in the study by Hoek and Robertson^25^ demonstrated the residual impact of decades of marketing by the tobacco industry, where white, slim cigarettes were associated with glamour, femininity and sophistication^25^. Younger participants and non-smokers however did not experience the same reaction, as they found many cigarettes interesting or attractive if they differed from their expectation of a ‘standard’ cigarette^24^. However, some changes received positive responses from most groups, such as the inclusion of gold bands or light colourations, which were associated with an increase in style and glamour^11,24,28^.

Dark colourations, however, were associated with sickness and dirtiness, and seen as a dissent to the desired persona of smokers^25^. This led to smokers reporting a reduction in the perceived enjoyment experienced from these cigarettes, as well as a perceived reduction in product quality^25^. Hoek and Robertson^25^ discussed this work extending on the Cue Consistency Theory, where specific designs are used to appeal to a certain population, often young women, and therefore the intentional design of dissuasive cigarettes could deter these populations from using tobacco products^25^. These findings were unsurprising, given the extensive internal research performed by tobacco companies^18,19^.

Smoker characteristics such as age, gender, and ethnicity have been shown to influence cigarette preference in other studies^41^, with the long and ultra-long cigarettes being popular amongst women, African Americans, those of a higher socioeconomic status, and those within the middle age (45 years) and older age groups^19,40^. This was thought to be a result of social, societal, and marketing forces within the United States, made more alarming by the perception of reduced harm of long and ultra-long cigarettes, and the substantially increased cotinine, urinary total 4-(methylnitrosamino)-1-(3-pyridyl)-1-butanonol (NNAL), and cadmium levels of these smokers^40,42,43^.

Implementing health warnings on cigarette sticks may encounter logistical barriers, particularly the inclusion of meaningful messages on a small surface area. Moodie et al.^30,31^ used only a single message ‘Smoking kills’, which is already a well-established health warning used on tobacco packaging^30^. The advantage of this message is that it is short and easily understood, allowing it to be placed in a variety of orientations on individual cigarettes, and in a large font size. However, one smoker responded that ‘You know smoking kills anyway’, and others reported it as being a lecturing message rather than being an informative or novel message^28,30^.

Moodie et al.^31^ also investigated the opinions of 12 packaging and marketing experts on the novel health messages introduced in Moodie et al.^30^, including an on-cigarette health warning. These experts described the message ‘Smoking Kills’ as powerful and effective, and could be easily incorporated on cigarettes using non-toxic, vegetable-based inks, which are already use on cigarette papers. They also described the increased exposure of smokers to the on-cigarette warning as opposed to pack-warnings, and the potentially significant psychological impact of the warning to both smokers and observers^44^.

Hassan and Shiu^29^ found that the more novel message ‘Minutes of life lost’, obtained the lowest attractiveness ratings, and yielded the highest change in quit intentions, after being viewed by the participants. This on-stick warning, which covered a significant surface area of the cigarette, may affect both smokers and non-smokers/casual smokers, through encouraging quit attempts and preventing cigarette experimentation, respectively. Cigarettes listing toxic constituents were considered not as off-putting as the ‘Minutes of life lost’ cigarettes, and may have had their effectiveness reduced by a lack of understanding of the impact of the chemicals listed^29^. Studies conducted in the UK, US, Australia, Canada, and Mexico showed that smokers are largely unaware of the toxic constituents of cigarettes and the harm they cause, thereby negating the effect of this warning^27,45,46^. This review found that the inclusion of health warnings on cigarettes: was particularly effective in changing the perceptions of participants, validated the decision to regulate cigarette stick appearance in Australia as part of the Tobacco Plain Packaging Act, and supports the decision of other FCTC signatories to begin standardising cigarette appearance.

Further research, with larger numbers and demographic profiles of participants, is needed to better evaluate the generalizable effects of unappealing visual cigarette attributes on smoker and non-smoker perceptions of smoking. This will give a better understanding of their efficacy in influencing smoking cessation and preventing smoking initiation. As adolescents (particularly women) are the primary targets for marketing strategies by tobacco companies, larger-scale evaluations of dissuasive colourations and cautionary health warnings within this specific population would also be of benefit. Focus groups and one-on-one interviews using modified cigarettes will likely retrieve the most comprehensive data, as opposed to online questionnaires that use photographs or illustrations, as thought processes underlying the perceptions of participants are more valuable than quantitative responses and the ranking of cigarettes. Widely recognised ‘danger’ symbols (such as the iconic ‘skull and crossbones’) might also be effective in supplementing text messages. Additional aspects of sensory appeal, such as taste and smell, and perceived cigarette strength can also contribute to misperceptions of cigarette harm, and could be altered to dissuade smokers and non-smokers from using tobacco products^24,26^. Alternative novel techniques for the communication of the harms of smoking that also require further research include pack inserts (currently used only in Canada), audio messages, and Quick Response (QR) codes^30,31,47,48^.

Limitations of this review include the small number of participants in many of the studies, as well as limited sample sizes. Most studies enrolled participants under 30 years old, all studies were set in Westernised countries, three studies enrolled only women, and four studies gathered perceptions from less than 100 participants. These issues make the generalization of results to a wider population difficult, such as to men, the middle aged and the elderly, and to less developed countries where public policy and perceptions of smoking may be different from developed countries. Lastly, none of the studies in this review was conducted in post-plain packaging environments, which would potentially strengthening the results, through enhancing dissuasive colours and health warnings after removal of attractive visual branding on the outer packaging. Further research is needed to identify the most effective physical modifications and health warnings in reducing cigarette smoking prevalence.

### Strengths and limitations of this study

- This systematic review investigates the impact of a novel extension to a tobacco control strategy that involves the implementation of visually unappealing attributes on individual cigarette sticks to reduce smoking appeal and increase perceptions of harm.
- Some insight is provided into the impact of a variety of visually unappealing cigarette stick attributes, although extrapolating these results to a population level is limited.
- Few articles were eligible for inclusion, with each assessing different visual attributes, and many also having small participant-sample sizes, often being restricted to young or female participants.

## CONCLUSIONS

Current written and illustrative health warnings and the plain packaging of tobacco products have served as an effective means of communicating health risks associated with smoking. There is, however, a need to further improve on quit rates and to prevent people from commencing smoking, especially adolescents, in order to reduce health risks and positively impact on population health. Thus, extending these interventions to affect the visual attributes of the cigarette may have additional benefits. This systematic review has identified and discussed the perceptions of physical cigarette design and health messages on cigarettes. Dissuasive visual attributes of cigarette sticks, such as larger dimensions and dark colouration, and the inclusion of health warnings on cigarette sticks may serve as an effective tobacco control method, potentially leading to a reduction in tobacco use.

## CONFLICTS OF INTEREST

Authors have completed and submitted the ICMJE Form for Disclosure of Potential Conflicts of Interest and none was reported.
